# (*RS*)-2-Oxo-4-(1-phenyl­ethyl­amino)-1,2-dihydro­quinoline-3-carb­oxy­lic acid

**DOI:** 10.1107/S1600536811043297

**Published:** 2011-10-29

**Authors:** Svitlana V. Shishkina, Igor V. Ukrainets, Elena V. Mospanova

**Affiliations:** a STC ‘Institute for Single Crystals’, National Academy of Sciences of Ukraine, 60 Lenina avenue, Kharkiv 61001, Ukraine; b National University of Pharmacy, 4 Blyukhera avenue, Kharkiv 61002, Ukraine

## Abstract

The mol­ecular structure of the title compound, C_18_H_16_N_2_O_3_, does not differ in the crystals of the racemic mixture, (I), and the pure enantiomer, (II). In their crystal structures, inversion dimers occur in (I) *via* N—H⋯O hydrogen bonds and infinite chains in (II) also *via* N—H⋯O hydrogen bonds.

## Related literature

For the *S* and *R* enanti­omers, see: Ukrainets *et al.* (2010 ▶). For bond lengths in related structures, see: Bürgi & Dunitz (1994 ▶).
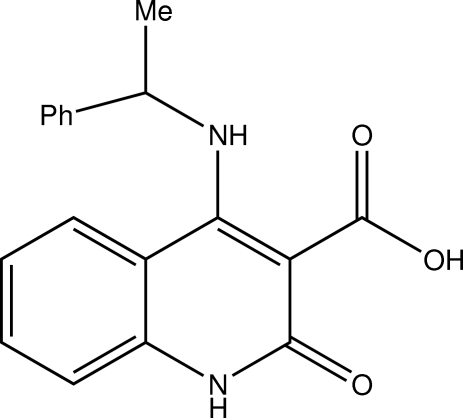


## Experimental

### 

#### Crystal data

C_18_H_16_N_2_O_3_
*M* *_r_* = 308.33
Monoclinic, 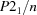

*a* = 14.612 (2) Å
*b* = 5.9750 (6) Å
*c* = 18.014 (2) Å
β = 110.814 (14)°
*V* = 1470.0 (3) Å^3^
*Z* = 4
Mo *K*α radiation
μ = 0.10 mm^−1^
*T* = 293 K
0.30 × 0.10 × 0.05 mm

#### Data collection

Oxford Diffraction Xcalibur 3 diffractometer
10943 measured reflections
2541 independent reflections
1174 reflections with *I* > 2σ(*I*)
*R*_int_ = 0.068

#### Refinement

*R*[*F* ^2^ > 2σ(*F* ^2^)] = 0.031
*wR*(*F* ^2^) = 0.033
*S* = 0.66
2541 reflections
221 parameters
H atoms treated by a mixture of independent and constrained refinement
Δρ_max_ = 0.10 e Å^−3^
Δρ_min_ = −0.10 e Å^−3^

### 

Data collection: *CrysAlis PRO* (Oxford Diffraction, 2005 ▶); cell refinement: *CrysAlis PRO*; data reduction: *CrysAlis RED* (Oxford Diffraction, 2005 ▶); program(s) used to solve structure: *SHELXTL* (Sheldrick, 2008 ▶); program(s) used to refine structure: *SHELXTL*; molecular graphics: *SHELXTL*; software used to prepare material for publication: *SHELXTL*.

## Supplementary Material

Crystal structure: contains datablock(s) I, global. DOI: 10.1107/S1600536811043297/jj2104sup1.cif

Structure factors: contains datablock(s) I. DOI: 10.1107/S1600536811043297/jj2104Isup2.hkl

Supplementary material file. DOI: 10.1107/S1600536811043297/jj2104Isup3.cml

Additional supplementary materials:  crystallographic information; 3D view; checkCIF report

## Figures and Tables

**Table 1:** Hydrogen-bond geometry (Å, °)

*D*—H⋯*A*	*D*—H	H⋯*A*	*D*⋯*A*	*D*—H⋯*A*
N1—H1N⋯O1^i^	1.038 (17)	1.794 (17)	2.8291 (15)	174.5 (14)
N2—H2N⋯O2	0.926 (14)	1.738 (14)	2.5849 (17)	150.4 (12)
O3—H3O⋯O1	0.943 (19)	1.59 (2)	2.4712 (15)	154.1 (18)

Symmetry code: (i) 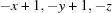.

**Fig. 1.**
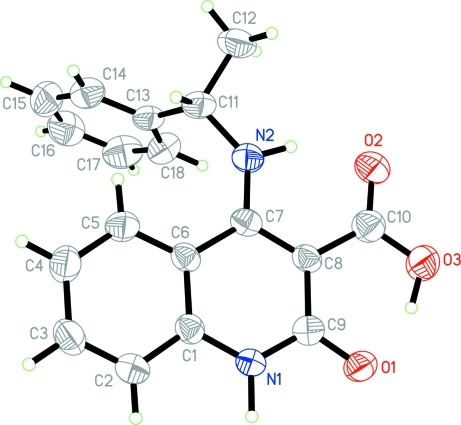
View of the title compound with atomic numbering. All atoms are shown with displacement ellipsoids drawn at the 50% probability level.

**Fig. 2.**
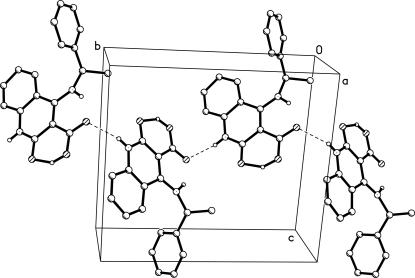
The packing of the pure enantiomer (II) in crystal phase. Hydrogen bonds are shown by dashed lines.

**Fig. 3.**
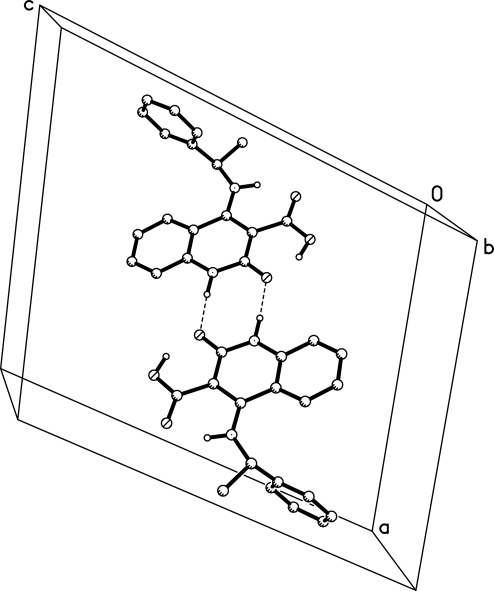
The packing of the title racemate (I) in crystal phase. Hydrogen bonds are shown by dashed lines.

## supplementary crystallographic information

### Comment

In the title compound, (I), the racemate of 2-oxo-4-(1-phenylethylamino)-1,2- dihydroquinoline-3-carboxylic acid reveals high analgesic activity. Compared to its pure S and *R* enantiomers, they are completely inactive (Ukrainets *et al.*, 2010). In this paper we compare the molecular and crystal structure of the racemate (I) with a previously studied structure of the pure enantiomer (II).  In the title compound (Fig. 1) the formation of two strong N2—H···O2 and O3—H···O1 intramolecular hydrogen bonds (Table 1) contributes to the coplanarity of the heterocycle, carboxyl, carbonyl groups and N2 atom all to be within 0.02 Å.  As a result a significant redistribution of the electron density occurs in the quinolone fragment: the O3—C10 and C8—C9 bonds are shortened (Table 1) as compared with their mean values of 1.362 Å and 1.455 Å (Bürgi & Dunitz, 1994). The O1—C9, O2—C10, and C7—C8 bonds are elongated (mean values are 1.210 Å for a C*sp*^2^= O bond and 1.418 Å for a C*sp*^2^= C*sp*^2^ bond). The substituent at the amino group has a *sp*- conformation. The C6—C7 bond (C11/N2/C7/C6 torsion angle = -1.6 (2)%A) is twisted slghtly allowing the methyl group to be *ap*- oriented relative to the C7—N2 bond (C7/N2/C11/C12 torsion angle = 171.3 (1)%A). The phenyl substituent is in a -*sc*-conformation relative to the C7—N2 bond and is twisted toward the N2—C11 bond (C7/N2/C11/C13 and N2/C11/C13/C18 torsion angles = -67.3 (2) %A and 36.3 (2) %A, respectively). The crystal structure of (I), therefore, differs significantly from that of (II).  In the pure enantiomer (II) infinite chains (Fig. 2) result from the formation of an N1—H···O2 intermolecular hydrogen bond (Ukrainets *et al.*, 2010). In the racemte, (I), centrosymmetric dimers (Fig. 3) are formed by a N1—H1N···O1 intermolecular hydrogen bond (Table 2).  This allows for Cg1—Cg1 π—π stacking interactions to be observed [centroid–centroid distance = 3.894 (1)Å^i^; *i* = 1-x, 2-y, -z; Cg1 = N1/C1/C6-C9].

### Experimental

2-Oxo-4-(1-phenylethylamino)-1,2-dihydroquinoline-3-carboxylic acid was synthesized using the published method (Ukrainets *et al.*, 2010). Yield 75%. *M*.p. 225–227° C.

### Refinement

H1N, H2N and H3O were located from by a Fourier map and refined isotropically. All of the remaining hydrogen atoms were placed in their calculated positions and then refined using the riding model with Atom—H lengths of 0.93Å (CH) or 0.96Å (CH_3_).  Isotropic displacement parameters for these atoms were set to 1.2 (CH) or 1.5 (CH_3_) times *U*_eq_ of the parent atom.

### Crystal data

**Table:**

C_18_H_16_N_2_O_3_	*F*(000) = 648
*M**_r_* = 308.33	*D*_x_ = 1.393 Mg m^−^^3^
Monoclinic, *P*2_1_/*n*	Melting point = 498–500 K
Hall symbol: -P 2yn	Mo *K*α radiation, λ = 0.71073 Å
*a* = 14.612 (2) Å	Cell parameters from 1534 reflections
*b* = 5.9750 (6) Å	θ = 3.0–32.0°
*c* = 18.014 (2) Å	µ = 0.10 mm^−^^1^
β = 110.814 (14)°	*T* = 293 K
*V* = 1470.0 (3) Å^3^	Needle, colourless
*Z* = 4	0.30 × 0.10 × 0.05 mm

### Data collection

**Table:**

Oxford Diffraction Xcalibur 3 diffractometer	1174 reflections with *I* > 2σ(*I*)
Radiation source: Enhance (Mo) X-ray Source	*R*_int_ = 0.068
graphite	θ_max_ = 25.0°, θ_min_ = 3.0°
Detector resolution: 16.1827 pixels mm^-1^	*h* = −17→17
ω scans	*k* = −7→7
10943 measured reflections	*l* = −20→21
2541 independent reflections	

### Refinement

**Table:**

Refinement on *F*^2^	Primary atom site location: structure-invariant direct methods
Least-squares matrix: full	Secondary atom site location: difference Fourier map
*R*[*F*^2^ > 2σ(*F*^2^)] = 0.031	Hydrogen site location: difference Fourier map
*wR*(*F*^2^) = 0.033	H atoms treated by a mixture of independent and constrained refinement
*S* = 0.66	*w* = 1/[σ^2^(*F*_o_^2^) + (0.0067*P*)^2^] where *P* = (*F*_o_^2^ + 2*F*_c_^2^)/3
2541 reflections	(Δ/σ)_max_ = 0.001
221 parameters	Δρ_max_ = 0.10 e Å^−^^3^
0 restraints	Δρ_min_ = −0.10 e Å^−^^3^

### Special details

**Table:**

Geometry. All e.s.d.'s (except the e.s.d. in the dihedral angle between two l.s. planes) are estimated using the full covariance matrix. The cell e.s.d.'s are taken into account individually in the estimation of e.s.d.'s in distances, angles and torsion angles; correlations between e.s.d.'s in cell parameters are only used when they are defined by crystal symmetry. An approximate (isotropic) treatment of cell e.s.d.'s is used for estimating e.s.d.'s involving l.s. planes.
Refinement. Refinement of *F*^2^ against ALL reflections. The weighted *R*-factor *wR* and goodness of fit *S* are based on *F*^2^, conventional *R*-factors *R* are based on *F*, with *F* set to zero for negative *F*^2^. The threshold expression of *F*^2^ > σ(*F*^2^) is used only for calculating *R*-factors(gt) *etc*. and is not relevant to the choice of reflections for refinement. *R*-factors based on *F*^2^ are statistically about twice as large as those based on *F*, and *R*- factors based on ALL data will be even larger.

### Fractional atomic coordinates and isotropic or equivalent isotropic displacement parameters (Å^2^)

**Table:**

	*x*	*y*	*z*	*U*_iso_*/*U*_eq_	
N1	0.54636 (9)	0.7456 (2)	−0.03766 (8)	0.0393 (3)	
H1N	0.4891 (13)	0.631 (2)	−0.0545 (9)	0.116 (7)*	
N2	0.77647 (10)	1.19200 (19)	0.02901 (9)	0.0456 (4)	
H2N	0.8164 (9)	1.153 (2)	0.0800 (9)	0.058 (5)*	
O1	0.61318 (7)	0.55549 (16)	0.07595 (6)	0.0498 (3)	
O2	0.84379 (7)	0.97876 (16)	0.16236 (6)	0.0615 (3)	
O3	0.76182 (8)	0.6926 (2)	0.18244 (7)	0.0613 (4)	
H3O	0.7056 (15)	0.610 (3)	0.1530 (12)	0.138 (9)*	
C1	0.54064 (10)	0.9229 (2)	−0.08811 (8)	0.0350 (4)	
C2	0.45947 (10)	0.9355 (2)	−0.15744 (9)	0.0447 (4)	
H2	0.4131	0.8214	−0.1703	0.054*	
C3	0.44735 (11)	1.1146 (2)	−0.20685 (9)	0.0485 (4)	
H3	0.3921	1.1251	−0.2527	0.058*	
C4	0.51788 (11)	1.2804 (2)	−0.18817 (9)	0.0490 (4)	
H4	0.5097	1.4034	−0.2216	0.059*	
C5	0.59936 (10)	1.2656 (2)	−0.12129 (9)	0.0448 (4)	
H5	0.6463	1.3781	−0.1104	0.054*	
C6	0.61435 (10)	1.0856 (2)	−0.06863 (8)	0.0340 (4)	
C7	0.69759 (10)	1.0588 (2)	0.00556 (8)	0.0344 (4)	
C8	0.69570 (10)	0.8825 (2)	0.05766 (9)	0.0351 (4)	
C9	0.61762 (11)	0.7222 (2)	0.03378 (10)	0.0378 (4)	
C10	0.77197 (12)	0.8569 (3)	0.13643 (10)	0.0472 (4)	
C11	0.80766 (10)	1.3854 (2)	−0.00571 (9)	0.0424 (4)	
H11	0.7558	1.4987	−0.0191	0.051*	
C12	0.89830 (10)	1.4790 (2)	0.05891 (8)	0.0595 (5)	
H12C	0.9503	1.3712	0.0714	0.089*	
H12B	0.8833	1.5102	0.1056	0.089*	
H12A	0.9183	1.6145	0.0404	0.089*	
C13	0.83092 (10)	1.3343 (2)	−0.07889 (9)	0.0379 (4)	
C14	0.81570 (10)	1.4954 (2)	−0.13665 (10)	0.0498 (4)	
H14	0.7851	1.6288	−0.1324	0.060*	
C15	0.84497 (12)	1.4625 (3)	−0.20070 (10)	0.0609 (5)	
H15	0.8341	1.5733	−0.2392	0.073*	
C16	0.88995 (12)	1.2670 (3)	−0.20764 (11)	0.0664 (5)	
H16	0.9103	1.2443	−0.2504	0.080*	
C17	0.90463 (12)	1.1050 (3)	−0.15065 (12)	0.0642 (5)	
H17	0.9351	0.9718	−0.1552	0.077*	
C18	0.87513 (11)	1.1362 (2)	−0.08708 (10)	0.0513 (4)	
H18	0.8849	1.0236	−0.0494	0.062*	

### Atomic displacement parameters (Å^2^)

**Table:**

	*U*^11^	*U*^22^	*U*^33^	*U*^12^	*U*^13^	*U*^23^
N1	0.0343 (8)	0.0428 (8)	0.0359 (10)	−0.0088 (7)	0.0063 (7)	0.0011 (6)
N2	0.0365 (9)	0.0539 (9)	0.0423 (11)	−0.0131 (7)	0.0091 (8)	0.0010 (7)
O1	0.0443 (7)	0.0459 (6)	0.0516 (8)	−0.0080 (5)	0.0075 (6)	0.0116 (6)
O2	0.0469 (8)	0.0690 (7)	0.0517 (8)	−0.0163 (6)	−0.0031 (6)	0.0055 (6)
O3	0.0458 (8)	0.0709 (8)	0.0538 (10)	−0.0083 (7)	0.0011 (7)	0.0206 (7)
C1	0.0342 (10)	0.0403 (10)	0.0311 (11)	−0.0008 (8)	0.0122 (8)	−0.0026 (8)
C2	0.0367 (11)	0.0486 (10)	0.0443 (12)	−0.0072 (7)	0.0090 (9)	−0.0016 (8)
C3	0.0385 (10)	0.0665 (11)	0.0356 (12)	−0.0035 (9)	0.0071 (8)	0.0007 (9)
C4	0.0402 (10)	0.0580 (11)	0.0468 (13)	0.0002 (9)	0.0133 (9)	0.0142 (8)
C5	0.0330 (10)	0.0503 (10)	0.0497 (13)	−0.0089 (8)	0.0132 (9)	0.0036 (8)
C6	0.0306 (9)	0.0414 (10)	0.0305 (11)	−0.0022 (8)	0.0113 (8)	−0.0044 (7)
C7	0.0276 (10)	0.0399 (9)	0.0373 (11)	−0.0021 (8)	0.0135 (8)	−0.0065 (8)
C8	0.0259 (9)	0.0428 (10)	0.0334 (11)	−0.0028 (7)	0.0066 (8)	−0.0019 (8)
C9	0.0356 (10)	0.0367 (10)	0.0415 (12)	0.0008 (8)	0.0143 (9)	−0.0011 (8)
C10	0.0426 (12)	0.0479 (12)	0.0490 (13)	−0.0006 (9)	0.0137 (10)	0.0017 (9)
C11	0.0357 (10)	0.0423 (9)	0.0499 (12)	−0.0100 (8)	0.0163 (9)	−0.0049 (8)
C12	0.0563 (11)	0.0621 (11)	0.0569 (13)	−0.0238 (9)	0.0161 (9)	−0.0139 (9)
C13	0.0334 (10)	0.0371 (10)	0.0440 (12)	−0.0090 (7)	0.0145 (8)	−0.0046 (8)
C14	0.0458 (11)	0.0454 (11)	0.0590 (13)	−0.0042 (7)	0.0195 (10)	−0.0003 (9)
C15	0.0609 (13)	0.0689 (13)	0.0548 (14)	−0.0091 (10)	0.0230 (10)	0.0084 (10)
C16	0.0680 (14)	0.0798 (15)	0.0593 (14)	−0.0109 (11)	0.0323 (11)	−0.0136 (11)
C17	0.0672 (13)	0.0517 (11)	0.0828 (16)	0.0006 (9)	0.0377 (12)	−0.0127 (11)
C18	0.0563 (12)	0.0433 (11)	0.0597 (13)	−0.0006 (9)	0.0273 (10)	0.0019 (9)

### Geometric parameters (Å, °)

**Table:**

N1—C9	1.3444 (17)	C7—C8	1.4177 (16)
N1—C1	1.3788 (16)	C8—C9	1.4334 (17)
N1—H1N	1.038 (17)	C8—C10	1.4679 (18)
N2—C7	1.3395 (15)	C11—C13	1.5051 (17)
N2—C11	1.4619 (16)	C11—C12	1.5251 (16)
N2—H2N	0.926 (14)	C11—H11	0.9800
O1—C9	1.2684 (15)	C12—H12C	0.9600
O2—C10	1.2252 (15)	C12—H12B	0.9600
O3—C10	1.3268 (17)	C12—H12A	0.9600
O3—H3O	0.943 (19)	C13—C14	1.3760 (16)
C1—C2	1.3854 (16)	C13—C18	1.3812 (16)
C1—C6	1.4000 (15)	C14—C15	1.3796 (19)
C2—C3	1.3626 (16)	C14—H14	0.9300
C2—H2	0.9300	C15—C16	1.3675 (19)
C3—C4	1.3825 (17)	C15—H15	0.9300
C3—H3	0.9300	C16—C17	1.3712 (19)
C4—C5	1.3629 (19)	C16—H16	0.9300
C4—H4	0.9300	C17—C18	1.3721 (19)
C5—C6	1.3986 (17)	C17—H17	0.9300
C5—H5	0.9300	C18—H18	0.9300
C6—C7	1.4614 (17)		
			
C9—N1—C1	123.76 (14)	O2—C10—O3	118.18 (16)
C9—N1—H1N	118.7 (9)	O2—C10—C8	124.05 (15)
C1—N1—H1N	117.3 (8)	O3—C10—C8	117.77 (14)
C7—N2—C11	134.34 (14)	N2—C11—C13	114.59 (12)
C7—N2—H2N	109.4 (8)	N2—C11—C12	106.35 (12)
C11—N2—H2N	116.2 (8)	C13—C11—C12	109.74 (12)
C10—O3—H3O	107.6 (12)	N2—C11—H11	108.7
N1—C1—C2	117.90 (14)	C13—C11—H11	108.7
N1—C1—C6	120.48 (14)	C12—C11—H11	108.7
C2—C1—C6	121.61 (14)	C11—C12—H12C	109.5
C3—C2—C1	120.16 (14)	C11—C12—H12B	109.5
C3—C2—H2	119.9	H12C—C12—H12B	109.5
C1—C2—H2	119.9	C11—C12—H12A	109.5
C2—C3—C4	119.44 (15)	H12C—C12—H12A	109.5
C2—C3—H3	120.3	H12B—C12—H12A	109.5
C4—C3—H3	120.3	C14—C13—C18	118.34 (14)
C5—C4—C3	120.65 (14)	C14—C13—C11	119.62 (14)
C5—C4—H4	119.7	C18—C13—C11	121.81 (14)
C3—C4—H4	119.7	C13—C14—C15	121.19 (15)
C4—C5—C6	121.79 (14)	C13—C14—H14	119.4
C4—C5—H5	119.1	C15—C14—H14	119.4
C6—C5—H5	119.1	C16—C15—C14	120.00 (16)
C5—C6—C1	116.28 (13)	C16—C15—H15	120.0
C5—C6—C7	125.69 (14)	C14—C15—H15	120.0
C1—C6—C7	117.98 (13)	C15—C16—C17	119.13 (17)
N2—C7—C8	116.72 (13)	C15—C16—H16	120.4
N2—C7—C6	124.51 (14)	C17—C16—H16	120.4
C8—C7—C6	118.77 (13)	C16—C17—C18	121.14 (16)
C7—C8—C9	119.89 (14)	C16—C17—H17	119.4
C7—C8—C10	122.05 (14)	C18—C17—H17	119.4
C9—C8—C10	118.06 (14)	C17—C18—C13	120.19 (14)
O1—C9—N1	117.89 (13)	C17—C18—H18	119.9
O1—C9—C8	123.34 (15)	C13—C18—H18	119.9
N1—C9—C8	118.75 (15)		
			
C9—N1—C1—C2	−175.26 (14)	C1—N1—C9—C8	−3.8 (2)
C9—N1—C1—C6	3.8 (2)	C7—C8—C9—O1	177.18 (13)
N1—C1—C2—C3	175.92 (13)	C10—C8—C9—O1	−2.7 (2)
C6—C1—C2—C3	−3.2 (2)	C7—C8—C9—N1	−1.2 (2)
C1—C2—C3—C4	1.6 (2)	C10—C8—C9—N1	178.91 (13)
C2—C3—C4—C5	0.4 (2)	C7—C8—C10—O2	−2.3 (2)
C3—C4—C5—C6	−0.9 (2)	C9—C8—C10—O2	177.57 (14)
C4—C5—C6—C1	−0.5 (2)	C7—C8—C10—O3	177.13 (13)
C4—C5—C6—C7	−177.97 (14)	C9—C8—C10—O3	−3.0 (2)
N1—C1—C6—C5	−176.49 (13)	C7—N2—C11—C13	−67.3 (2)
C2—C1—C6—C5	2.57 (19)	C7—N2—C11—C12	171.26 (14)
N1—C1—C6—C7	1.15 (19)	N2—C11—C13—C14	149.27 (13)
C2—C1—C6—C7	−179.79 (13)	C12—C11—C13—C14	−91.19 (15)
C11—N2—C7—C8	179.24 (15)	N2—C11—C13—C18	−36.34 (18)
C11—N2—C7—C6	−1.6 (2)	C12—C11—C13—C18	83.21 (15)
C5—C6—C7—N2	−7.6 (2)	C18—C13—C14—C15	−1.0 (2)
C1—C6—C7—N2	175.04 (13)	C11—C13—C14—C15	173.62 (14)
C5—C6—C7—C8	171.59 (13)	C13—C14—C15—C16	0.0 (2)
C1—C6—C7—C8	−5.81 (18)	C14—C15—C16—C17	0.5 (2)
N2—C7—C8—C9	−174.91 (12)	C15—C16—C17—C18	−0.1 (3)
C6—C7—C8—C9	5.87 (19)	C16—C17—C18—C13	−0.8 (3)
N2—C7—C8—C10	4.98 (19)	C14—C13—C18—C17	1.4 (2)
C6—C7—C8—C10	−174.23 (13)	C11—C13—C18—C17	−173.10 (15)
C1—N1—C9—O1	177.72 (12)		

### Hydrogen-bond geometry (Å, °)

**Table:**

*D*—H···*A*	*D*—H	H···*A*	*D*···*A*	*D*—H···*A*
N1—H1N···O1^i^	1.038 (17)	1.794 (17)	2.8291 (15)	174.5 (14)
N2—H2N···O2	0.926 (14)	1.738 (14)	2.5849 (17)	150.4 (12)
O3—H3O···O1	0.943 (19)	1.59 (2)	2.4712 (15)	154.1 (18)

Symmetry codes: (i) −*x*+1, −*y*+1, −*z*.
